# Development of nsP2 protease based cell free high throughput screening assay for evaluation of inhibitors against emerging Chikungunya virus

**DOI:** 10.1038/s41598-018-29024-2

**Published:** 2018-07-17

**Authors:** Amrita Saha, Badri Narayan Acharya, Raj Priya, Nagesh K. Tripathi, Ambuj Shrivastava, M. Kameswara Rao, Pooja Kesari, Manju Narwal, Shailly Tomar, Sameer S. Bhagyawant, Manmohan Parida, Paban Kumar Dash

**Affiliations:** 1 Virology Division, Defence Research & Development Establishment, Gwalior, 474002 India; 2 Synthetic Chemistry Division, Defence Research & Development Establishment, Gwalior, 474002 India; 3 Bioprocess Technology Division, Defence Research & Development Establishment, Gwalior, 474002 India; 4 Pharmacology & Toxicology Division, Defence Research & Development Establishment, Gwalior, 474002 India; 5 Department of Biotechnology, Indian Institute of Technology Roorkee, Roorkee, 247667 India; 6 School of Studies in Biotechnology, Jiwaji University, Gwalior, India

## Abstract

Chikungunya virus has emerged as one of the most important global arboviral threats over the last decade. Inspite of large scale morbidity, with long lasting polyarthralgia, so far no licensed vaccine or antiviral is available. CHIKV nsP2 protease is crucial for processing of viral nonstructural polypeptide precursor to release enzymes required for viral replication, thus making it a promising drug target. In this study, high cell density cultivation (HCDC) of *Escherichia coli* in batch process was carried out to produce rCHIKV nsP2pro in a cost-effective manner. The purified nsP2pro and fluorogenic peptide substrate have been adapted for fluorescence resonance energy transfer (FRET) based high throughput screening (HTS) assay with Z’ value and CV of 0.67 ± 0.054 and <10% respectively. We used this cell free HTS system to screen panel of metal ions and its conjugate which revealed zinc acetate as a potential candidate, which was further found to inhibit CHIKV in Vero cells. Scale-up process has not been previously reported for any of the arboviral nonstructural enzymes. The successful scale-up method for viral protease together with a HTS assay could lead to the development of industrial level large-scale screening platform for identification of protease inhibitors against emerging and re-emerging viruses.

## Introduction

Chikungunya virus (CHIKV) is one of the most important arboviruses and is primarily transmitted by *Aedes* mosquitoes^1^. Though CHIKV was isolated almost five decades ago, however the last decade witnessed numerous major outbreaks spanning different continents. It is now reported from both old and new world and is recognized as a global public health concern. The incursion of African genotype into Asia and Asian genotype into Americas led to massive unprecedented outbreaks in naïve population^2,3^. CHIKV infection in human may last for 1–10 days and the clinical spectrum ranges from acute infection to severe complications^4–7^. The higher number of severe cases indicates an urgent need to develop safe and effective medical countermeasures against CHIKV. Currently there is no licensed vaccine or therapeutics available against CHIKV.

CHIKV belongs to the family *Togaviridae*, genus *Alphavirus*^1,8^. CHIKV has a single-stranded positive-sense RNA genome which encodes two polyproteins viz non-structural and structural. During viral replication, non-structural polyprotein is processed into four nonstructural proteins (nsP1, nsP2, nsP3, and nsP4) responsible for viral replication and evasion from the host defence mechanisms. In addition structural polyprotein is processed into various proteins (C, E3, E2, 6K, E1) that are involved in viral particle assembly^9^. Currently, targeting viral enzymes that are crucial for viral replication is believed to be an attractive strategy for development of antiviral therapy. The CHIKV nsP2 protein is a multifunctional protein. The C-terminal domain of nsP2 acts as papain-like cysteine proteases (nsP2pro) with conserved catalytic dyad (Cys and His)^9,10^. The nsP2pro domain is responsible for cytoplasmic cleavages of polypeptide nsP123/1234 at junctions between nsP1/nsP2, nsP2/nsP3, and nsP3/nsP4 (AGA/GII, AGC/APS and AGG/YIF respectively)^9,10^. Thus, the pivotal role of nsP2pro in CHIKV replication makes it a potential antiviral target, as disruption of protease activity is lethal for virus. Inhibition of viral protease has already been successfully exploited for development of antiviral compounds against Human immunodeficiency virus (HIV) and Hepatitis C virus^11–13^. These successes have generated immense interest for development of protease inhibitors against other viral infections.

Though, virus-encoded proteases have emerged as an important target for antiviral intervention, but challenges do exist to develop potential lead molecules against viral protease. One of these challenges includes lack of an efficient assay system for screening inhibitors in HTS format. So far, few candidates were obtained through cell-based assays^14,15^. The cell based assay has been low to medium throughput due to involvement of multiple steps. Moreover, their exact targets remain unknown and specificity is relatively low. Thus, development of a sensitive and robust HTS assay is very crucial for screening potent candidates against specific targets in the near future.

The production of recombinant protease has been crucial to the development of biochemical HTS assay. The ability to screen large number of small molecule compound library in HTS platform is dependent on ability to produce large quantities of biologically active pure enzyme. Expression and purification of CHIKV nsP2pro has been problematic due to its low solubility, stability and poor yield etc^16,17^. There are many different eukaryotic and prokaryotic systems that can be applied to the production of enzymatically active proteases. Bacterial expression system is usually preferred, due to their ease of use and inexpensive fermentation and scale-up costs to meet greater demands^18–20^. Since CHIKV nsP2pro domain does not require post translational modification, therefore it is a good candidate for production in bacterial system.

There is a need to optimize suitable conditions for scale up of CHIKV nsP2pro for laboratory as well as industry level for enzyme production in large scale. Some of the important parameters for enhancing protein production are medium composition, cultivation conditions, inducer concentration, induction time and duration, and mode of cultivation that can be optimized to get recombinant protein with high yield^19,21^. The purification of recombinant enzyme is necessary with minimum possible steps to avoid loss of activity or degradation and achieve the required level of purity for therapeutic studies^22,23^. In general, scale up cultivation process at the optimal condition increases the expression and production level of enzyme to many times in comparison to shake flask. Thus, successful scalable production is crucial for the development of cost-effective HTS assay platform avoiding batch to batch variation.

Fluorescence has emerged as the format of choice for the development of wide variety of HTS screening assays, offering the advantage of a mix and measure homogeneous format^24^. Thus, based on the ability of the rCHIKV nsP2pro to cleave synthetic fluorogenic peptides containing the enzyme’s natural cleavage site, a FRET-based HTS assay platform for antiviral compound screening was developed in this study that can be readily applied to large-scale screening of small-molecule libraries. FDA approved drug zinc acetate (ZnAc) was found effective in this study in inhibiting rCHIKV nsp2pro, which was further evaluated in Vero cells to inhibit CHIKV replication.

## Results

### Cloning, expression and Solubility optimization

A 1005 bp segment encoding CHIKV nsP2pro was amplified using viral RNA as template by two step RT-PCR. The purified CHIKV nsP2pro amplicon was cloned in pET41a vector in frame with glutathione S-transferase (GST) and 6xHis at N-terminal, under T7 promoter. Screening of selected clones by sequencing revealed correct reading frame. SDS-PAGE analysis of the expressed protein on induction with 1 mM IPTG, showed expression of ~71 kDa CHIKV nsP2pro fusion protein with a dual (His_6_-GST) affinity tag. According to the densitometric analysis of bands on SDS-PAGE gel, under standard induction conditions (37 °C, 1 mM IPTG, 4 h), the recombinant CHIKV nsP2pro was found mainly in insoluble inclusion bodies (~80%), whereas, a very small fraction of protein remained in soluble form (~20%). Optimization of inducer concentration, temperature and induction time (18 °C, 0.5 mM IPTG, overnight) resulted in higher expression level of soluble CHIKV nsP2pro (~75%).

### Shake flask cultivation and purification

Purification of soluble fraction of recombinant protein from 1 L shake flask cell pellet lysate was performed by exploiting the 6xHis tag by using Ni^2+^-NTA affinity chromatography under native conditions. The protein was eluted by using 250 mM imidazole and was found to be >95% pure by gel analysis. GST protein was also expressed and purified under native condition to be used as a control to confirm digested GST moiety size and as a negative control for protease assay. The yield of recombinant CHIKV nsP2pro under native condition was estimated to be 6 mg/L of shake flask culture. The reactivity of purified CHIKV nsP2pro was evaluated using three different antibodies i.e. rabbit anti-nsP2 polyclonal, anti-His monoclonal or anti-GST monoclonal antibody. The recombinant fusion protein showed the affinity to react with all three antibodies and a specific band of ~71 kDa representing recombinant CHIKV nsP2pro fusion protein was detected. To monitor the proper enterokinase cleavage of the fusion protein, the immuno-reactivity of the cleaved fraction was also confirmed by western blot analysis using anti-nsP2 polyclonal antibody with a band size of ~39 kDa. The yield of the enterokinase cleaved purified protein was estimated to be 1 mg/L of the shake flask culture (Supplementary Fig. S1).

### Cultivation in Bioreactor and purification

**Figure 1:**
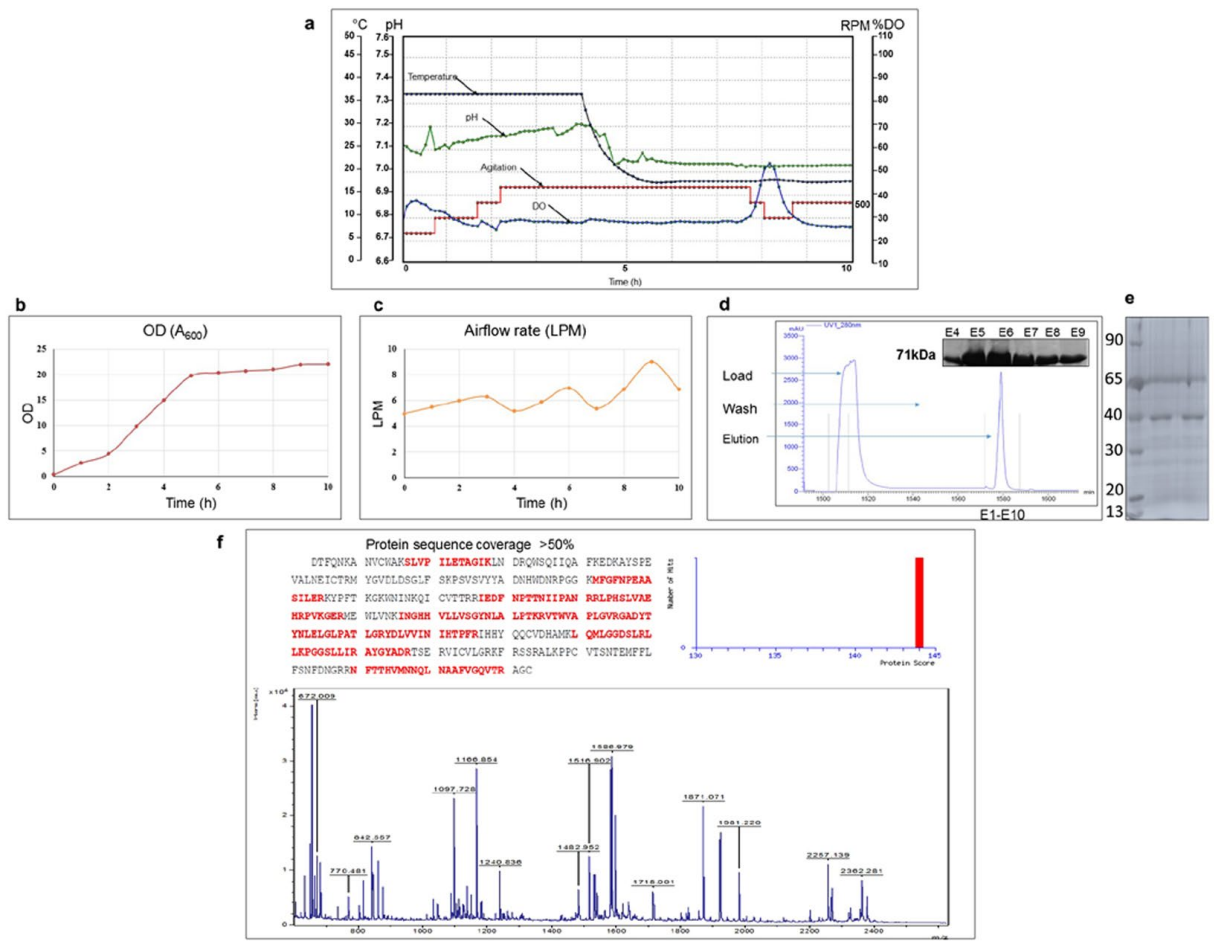
(**a**) Real-time profile of batch fermentation of *E*.*coli* BL21 (DE3) [pET41a + CHIKV nsP2pro] for expression of CHIKV nsP2pro. Culture was grown in Batch mode in 2XLB media with 1% glycerol and induced with 0.5 mM IPTG. Time profile for agitation (RPM), dissolved oxygen (%), temperature (°C) and pH; (**b**) OD (A_600_); (**c**) Airflow rate (L/min). (**d**) Chromatogram of affinity chromatography for purification of CHIKV nsP2pro fusion protein (71 kDa). (**e**) SDS–PAGE analysis of purified nsP2pro (39 kDa) after tag removal. (**f**) Purified protein band was cut out, digested with trypsin and analysed further with mass spectrometry analysis. MALDI-TOF/MS spectrum of CHIKV nsP2pro protein tryptic digests in reflectron mode using HCCA as matrix.

Different cultivation media like Luria Bertani (LB), Terrific Broth (TB) and Super Broth (SB) with some modifications were tried at small scale. The 2XLB with 1% glycerol was found to be best among all with respect to overall yield of a bioactive enzyme. The conditions optimized at shake flask level were further scaled up to ∼5 L culture in 7 L capacity bioreactor. Thus, for batch cultivation, cells were propagated in 2XLB broth supplemented with 1% glycerol and at an OD_600_ of 20, the culture was induced with 0.5 mM IPTG. In batch run, during the growth period, the dissolved oxygen (DO) level was maintained at >20% using agitation speed up to 600 rpm and air flow rate was maintained at 4–9 L/min along with supplying pure oxygen whenever it was required. Initially, DO level was >30% and it started declining during the course of growth of cells. Figure 1a–c shows the growth profile of the batch fermentation process. This study yielded ~22 g/L (>100 g/batch) of wet cell paste that was frozen at −80 °C till further use. Protease purified from long term stored pellet did not reveal any loss of yield and activity (Supplementary Fig. S1). The final dry cell weights were 1.2 and 7.2 g/L in shake flask and bioreactor respectively. Purification resulted in ~62 mg/L of nsP2pro fusion protein and 6 mg/L untagged nsP2pro (Fig. 1d,e). Purified protein was further characterized by MALDI-TOF analysis. The trypsin digested fusion protein gave multiple peptide peaks, 18 peptides for CHIKV nsP2pro and 14 peptides for GST were detected with the help of MASCOT search using NCBI database (Fig. 1f).

### Protease assay

**Figure 2:**
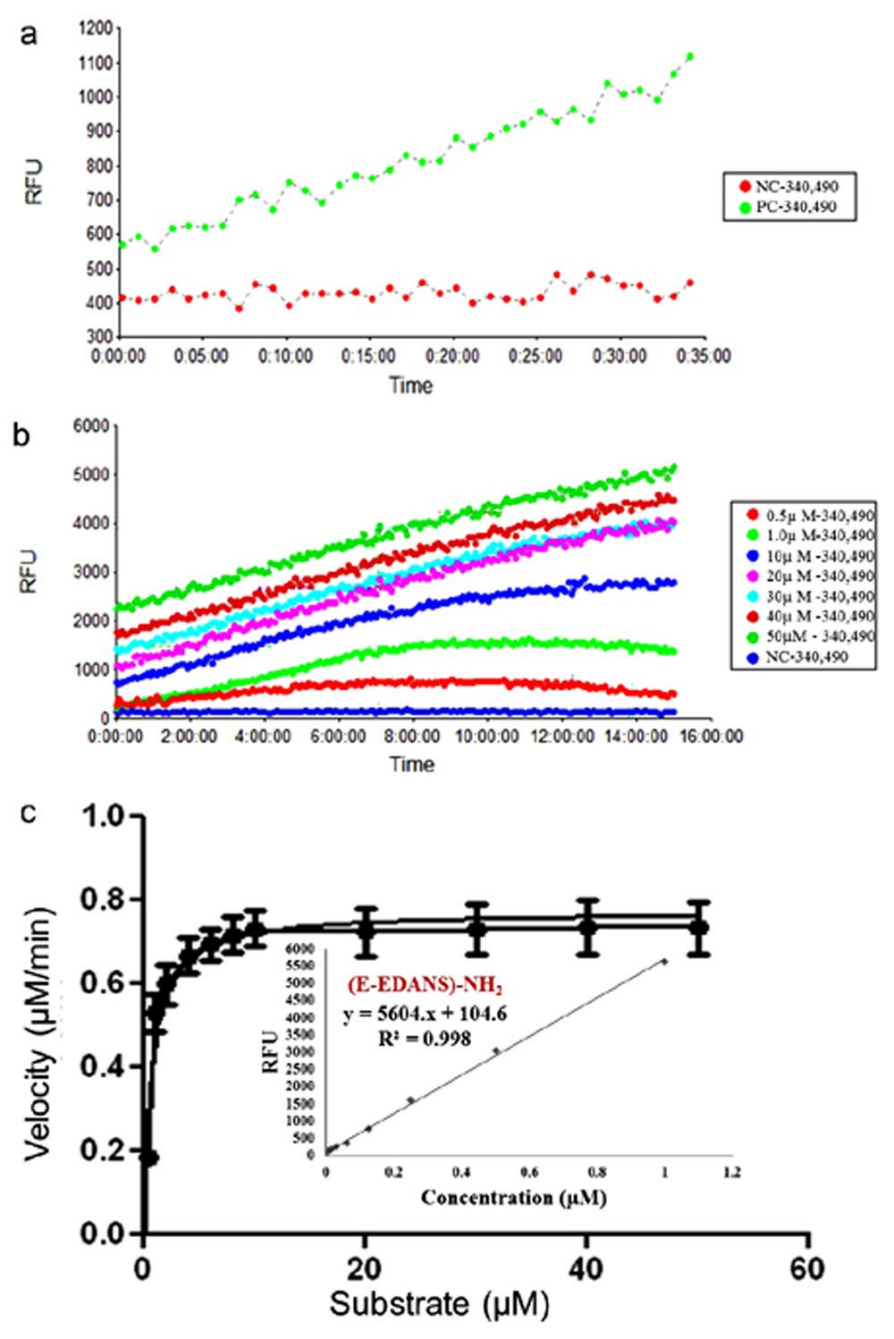
(**a**) A FRET-based protease assay has been developed for the determination of CHIKV nsP2pro activity. The donor EDANS (fluorophore) at C-terminal of octapeptide substrate produces the signal upon excitation that gets quenched by DABCYL (quencher) at N-terminal and thus shows FRET. In the presence of rCHIKV nsP2pro, the cleavage takes place and the EDANS and DABCYL gets separated from each other, which results in the reduction of FRET signal. Thus, control reaction with protease and substrate showed an increase in fluorescence unit (FU) with time (positive control, PC). A negative control (NC) reaction was also performed without enzyme, which did not showed change in the fluorescence intensity. In the presence of CHIKV nsP2pro inhibitors, the FRET should remain unaltered with loss of signal. (**b**) Real-time profile of proteolytic assay with different substrate concentrations ranging from 0.5–50 μM, using 5 μM protease was performed and the fluorescence was measured at different time points every 5 min till 15 h and the increase in fluorescence was observed. Background fluorescence (NC) was subtracted for clarity of comparison. (**c**) Reaction velocity (μM/min) calculated with the help of standard curve was plotted over different range of substrate concentration (means of three independent experiments performed in triplicate).

Initially, we used fluorescence intensity (FI) based tripeptide substrates, *t*-butyl-oxycarbonyl-Ala-Gly-Gly-7-methoxycoumarin-4-acetyl (Boc-AGC-MCA) and *t*-butyl-oxycarbonyl-Ala-Gly-Gly-7–amino-4-methylcoumarin (Boc-AGC-AMC) for the *trans*-proteolytic activity assay of CHIKV nsP2pro. Although, fluorescence unit of test reaction with these substrate were found higher than the background signal but a proper enzyme curve with time was not obtained (Supplementary Fig. S2). Subsequently, a highly sensitive FRET-based octapeptide substrate (DABCYL-Arg-Ala-Gly-Gly-Tyr-Ile-Phe-Ser-(Glu-EDANS)-NH_2_) was used for determining the activity of CHIKV nsP2protease. The active CHIKV nsP2protease exhibits the efficient cleavage of the substrate peptide, which separates fluorophore, 5-[(2-aminoethyl) amino] naphthalene-1-sulfonic acid (EDANS) and quencher, 4-[(4-(dimethylamino) phenyl-azo] benzoic acid (DABCYL) resulting in the increased fluorescence signal. Fluorescence profile for the hydrolysis of the FRET-based substrate with time is depicted in Fig. 2a. A control reaction was also performed using the same reaction without enzyme, which did not show change in the fluorescence intensity. Finally, experiments were carried out to estimate kinetic parameters of CHIKV nsP2pro by carrying out assay with increasing substrate concentration (Fig. 2b). Enzyme kinetic constant K_m_ and V_max_ derived were 0.98 ± 0.135 µM and 0.77 ± 0.024 µM min^−1^ respectively (Fig. 2c). Therefore, a proper reaction velocity saturation curve was obtained using longer FRET-based peptide substrate. Further, purified mutated C478A CHIKV nsP2pro was used as a negative control. C478A CHIKV nsP2pro was not able to cleave the substrate efficiently indicating that the C478 is crucial for nsP2pro activity. (Supplementary Fig. S3).

### Optimal reaction conditions and the effects of protease inhibitors

**Figure 3:**
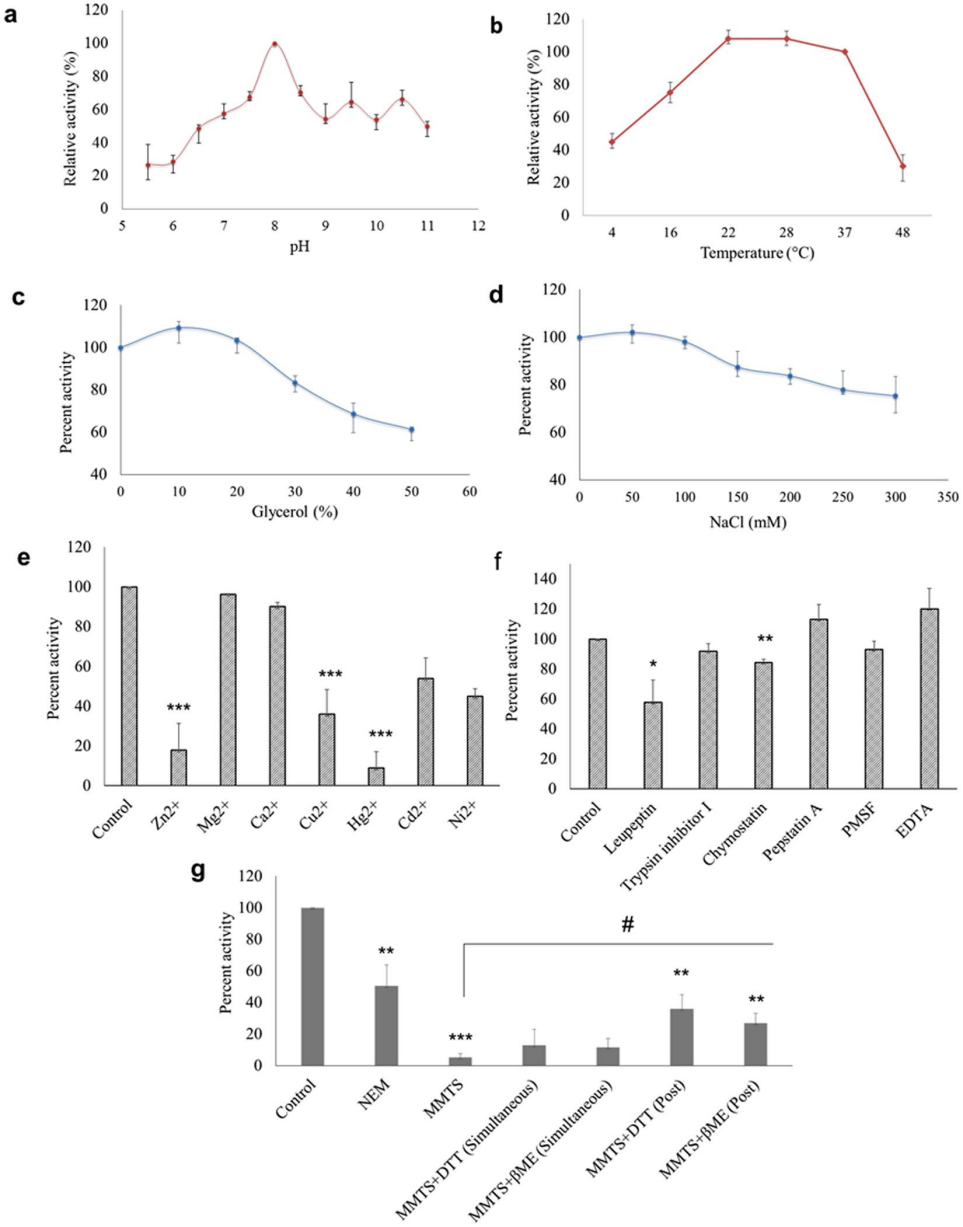
Optimal reaction conditions and effects of protease inhibitors for the processing activity of CHIKV nsP2pro (**a**) Effects of pH on protease activity. CHIKV nsP2pro and substrate were incubated in with various buffers of pH ranging from 5.5–11, [2-(N-morpholino) ethanesulfonic acid (MES buffer) for pH 5.5, 6.0; BisTris Propane buffer for pH 6.5, 7.0, 7.5, 8.0, 8.5, 9.0, 9.5 and N-cyclohexyl-3-aminopropanesulfonic acid (CAPS buffer) for pH 10.0, 10.5, 11 were used]. The relative activity was calculated at different pH by taking activity at pH 8.0 as 100%. (**b**) Effects of temperature (4°–48 °C), the relative activity were calculated by taking activity at 37 °C as 100%. (**c**) Effect of glycerol and (**d**) NaCl concentration on protease activity. CHIKV nsP2pro and substrate were incubated in a buffer of 20 mM BisTrisPropane (pH 8.0) containing the indicated concentrations (0, 50, 100, 150, 200, 250 and 300 mM) of NaCl. (**e**) Effects of various divalent cations (2 mM) on protease activity. (**f**) Effect of various universal protease inhibitors. CHIKV nsP2pro and substrate were incubated in the 20 mM BisTrisPropane (pH 8.0) with each of the indicated inhibitor. (**g**) Effects of thiol protease inhibitors (NEM and MMTS) on protease activity and the addition of DTT or β-mercaptoethanol (β-ME), restored the enzyme activity inhibited by MMTS when added simultaneously or 1 h post enzyme-substrate reaction. The standard errors of the means of results from three independent experiments are shown. The asterisk indicates statistical significance (*p < 0.05, **p < 0.01, *** < 0.001) with respect to control (^#^*p* value indicates significance with respect to MMTS).

Buffer and assay conditions were optimized for protease assay. The optimal pH was found to be 8, and lower pH impaired nsP2pro activity significantly in comparison to higher pH (Fig. 3a). The optimal temperature for protease activity was found to be 22–28 °C (Fig. 3b). The effect of glycerol (anti-chaotropic agent) was also observed. At 10–20% glycerol concentration slight increase in protease activity was seen. However, further increase in glycerol concentration resulted in the decrease of protease activity (Fig. 3c). The results with different Na^+^ concentrations indicated that the optimal concentration was around 50–100 mM and that the enzyme is relatively insensitive to Na^+^ (Fig. 3d). Effect of different divalent cations like Ca^2+^, Cd^2+^, Cu^2+^, Hg^2+^, Mg^2+^, Ni^2+^ and Zn^2+^ were studied on activity of nsP2pro. Although Mg^2+^ or Ca^2+^ did not affected protease activity however, Cu^2+^ (*p* value = 0.0005), Zn^2+^ (*p* value = 0.0002), and Hg^2+^ (*p* value = 5.0 × 10^−5^) decreased protease activity remarkably (Fig. 3e). Further, the effect of different universal protease inhibitors was also tested. The enzyme was completely resistant to the inhibitors of serine proteases (PMSF, trypsin protease inhibitor I), aspartic proteases (pepstatin) and metalloproteases (EDTA). It was moderately inhibited by cysteine protease inhibitor (leupeptin; *p* value = 0.05) followed by chymostatin (*p* value = 0.008) (Fig. 3f). The addition of sulfhydryl reagents such as N-ethylmaleimide (NEM; *p* value = 0.002) and methyl methanethiosulfonate (MMTS; *p* value = 7.1 × 10^−7^), almost completely inhibited the protease activity of rCHIKV nsP2pro. Moreover, the addition of DTT or β-mercaptoethanol (β-ME), partially restored the enzyme activity inhibited by MMTS, indicating that Cys residue in the catalytic site of CHIKV nsP2pro is important for the proteolytic activity (Fig. 3g).

### Validation of FRET assay for HTS

**Figure 4:**
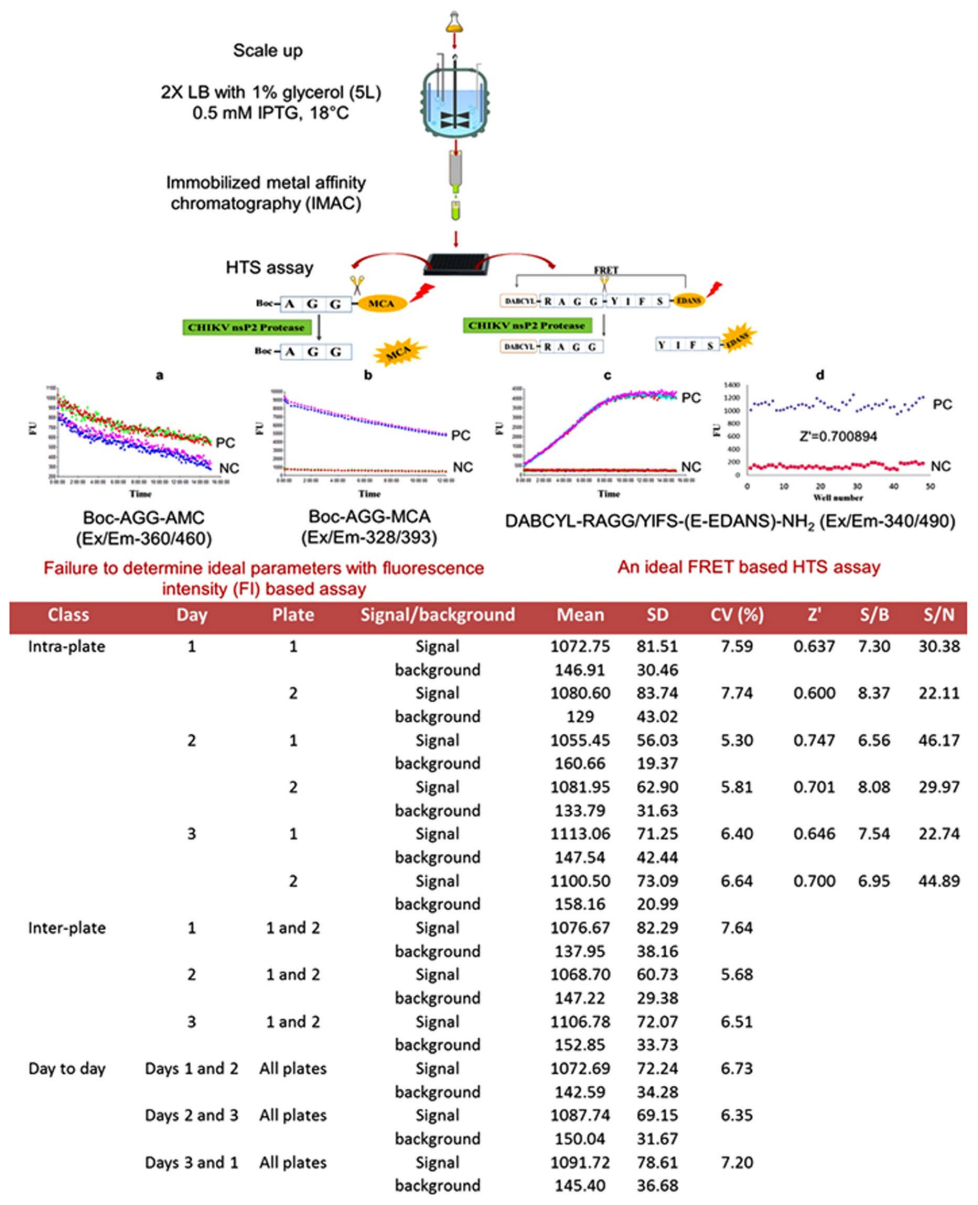
A schematic representing an experimental outline to determine ideal parameters to develop a sensitive and robust high throughput screening assay for screening small molecule inhibitors of the CHIKV nsP2pro. For development of HTS assay two different fluorescence-based approaches have been used to measure protease activity i.e., FI and FRET-based. (**a**–**c**) Real time profile of time course of product formation for FI and FRET based assay have been illustrated. The Z’ factor value > 0.5 has been considered as an indicator of an authentic HTS assay. (**d**) The scatter plot shows the positive control (diamond shaped) and negative control (square shaped) data for the Z’ factor calculation. Data shown here is representative of one of the intra-plate assay, using FRET-based substrate. Statistical analysis of data is tabulated which indicates mean, SD, CV, Z’-factor, S/B and S/N for each 96-well plate having 48 replicates of positive control (signal) and negative control (background) for intra-plate whereas mean, standard deviation (SD) and CV were calculated for inter-plate and day to day variability [Z’ = 1 − 3*(SD_signal_ + SD_background_)/(Mean_signal_ − Mean_background_), CV = SD_signal_/Mean_signal_ × 100, S/B = Mean_signal_/Mean_background_ and S/N = Mean_signal_ − Mean_background_/SD_background_].

The assay was validated statistically by determining the Z’ factor. The Z’ factor for the CHIKV nsP2pro was calculated to be 0.67 ± 0.054 using the FRET-based peptide substrate based on signal and background controls evaluated on a given day. In addition, intra-plate, inter-plate and day-to-day variability calculated was CV < 10% and signal-to-background ratio (S/B) obtained was >5 as evaluated on a given day, together these statistical parameters confirm the high sensitivity, specificity and efficiency of the assay (Fig. 4).

### Inhibition assay

**Figure 5:**
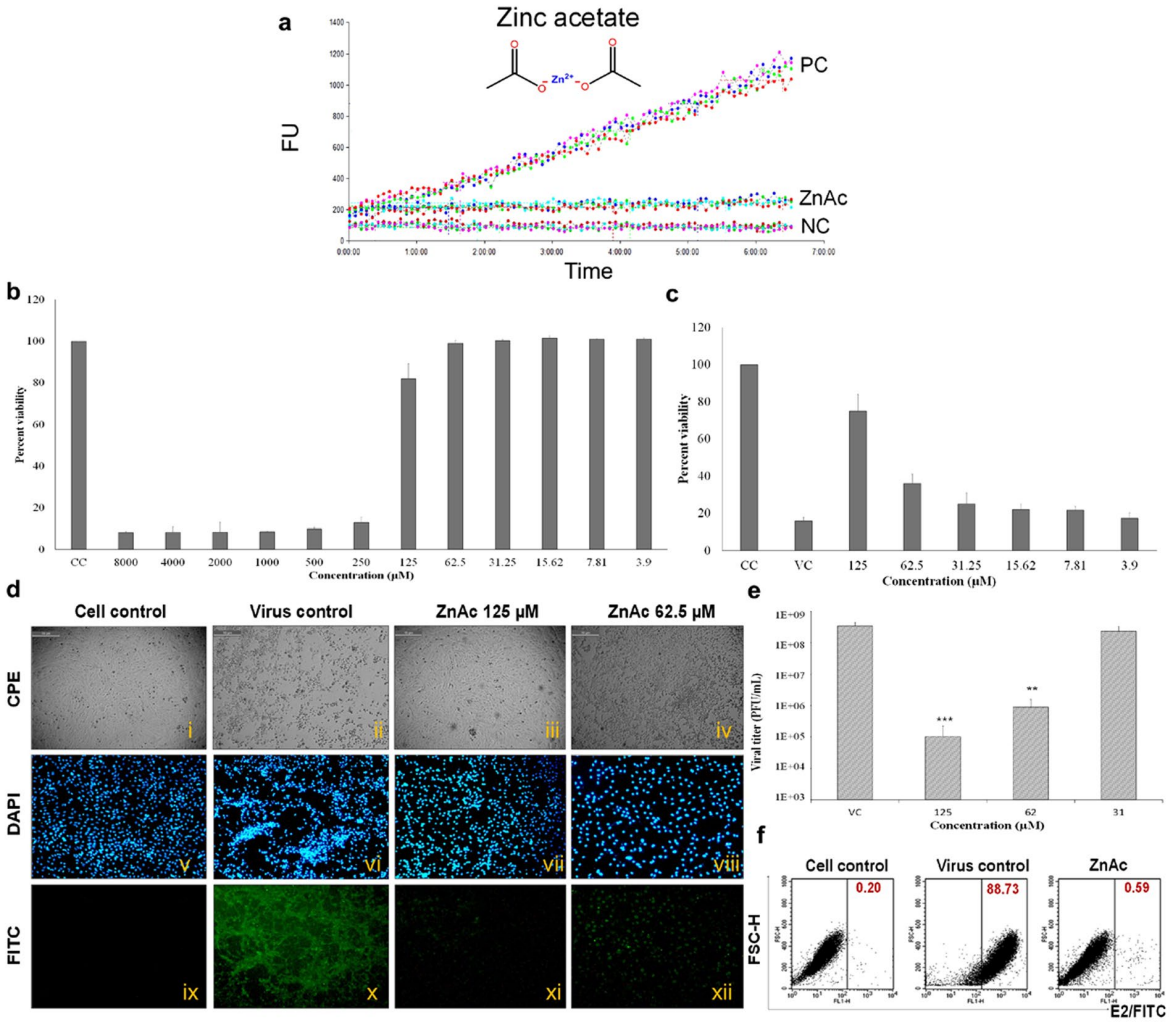
(**a**) Inhibition of rCHIKV nsP2pro activity by ZnAc as determined by FRET-based protease assay (2 µM substrate, 2 mM ZnAc, 20 mM BisTrisPropane pH 8.0 as assay buffer in 100 µl reaction volume at 25 °C for ~6 h). Real time profile monitored by fluorescence plate reader shows loss of signal indicating inhibition of rCHIKV nsP2pro by ZnAc. Toxicity and antiviral activity of ZnAc: (**b**) Cell viability assay (MTT assay) was performed to evaluate cytotoxicity of ZnAc on Vero cells. No significant cellular cytotoxicity was seen at 125 µM concentration; (**c**) Vero cells were infected with CHIKV at MOI 1 followed by addition of ZnAc post 2 h virus adsorption, MTT assay was performed 48 hpi to determine the percent viability after treatment; (**d**) Microscopic images (i-iv) showing CPE at 36 hpi after respective treatment; (v-xii) immunofluorescence assay was performed at 24 hpi, green fluorescence indicates the virus load as assessed with anti-CHIKV E2 mAb and secondary antibody conjugated with FITC and blue fluorescence indicates the nuclear staining with 4′,6-diamidino-2-phenylindole (DAPI) at 10× . Data shown here is representative of one of the three experimental repeats. (**e**) Cell supernatant was collected at 48 hpi and virus titer was assessed by plaque assay. Data represents the mean ± standard deviation of five independent experiments. The asterisk indicates statistical significance (*p < 0.05, **p < 0.01, *** < 0.001), calculated for the relative titer of infectious CHIKV. (**f**) Representative flow cytometry dot plot analysis of Vero cells that were left untreated (Cells + DMSO) or were further challenged with virus at MOI 0.01 (Virus + DMSO) or virus infected cells treated with 100 µM ZnAc for 24 h. The percentages of positive cells are indicated in respective quadrant. Data are representative of two independent experiments.

Inhibition of rCHIKV nsP2pro by ZnAc was measured in FRET-based assay. The cleavage of the fluorogenic substrate by nsP2pro was almost completely inhibited by 2 mM ZnAc, as reaction did not show change in the fluorescence intensity when monitored up to 6 h through fluorescence plate reader (Fig. 5a).

### Antiviral assay

In order to determine the cytotoxic effect of ZnAc before subsequent assays, the maximum nontoxic dose (MNTD) was determined on Vero cells by MTT assay. The MNTD value of ZnAc was found to be 125 µM that showed more than 85% cell viability (Fig. 5b). Antiviral activity was assessed by performing MTT assay on CHIKV infected Vero cells in the presence of various concentrations of ZnAc at 48 hpi, indicating >75% cell viability at 125 µM concentration (Fig. 5c). The virus induced cytopathic effect (CPE) was recorded at 36 hpi, showing inhibition in a dose-dependent manner (Fig. 5d). Antiviral activity of ZnAc through plaque reduction assay showed that CHIKV load was reduced significantly (*p* value = 8.1 × 10^−5^) by 99.97% (>3 log reduction) following treatment with 125 µM ZnAc at 48 hpi. CHIKV titer after treatment with 125 µM, 62.5 µM and 31.25 µM ZnAc were 1.02 × 10^5^, 8.89 × 10^5^ and 2.33 × 10^8^ PFU/ml respectively at 48 hpi, compared to 3.45 × 10^8^ PFU/ml in virus control (Fig. 5e). The immunofluorescence assay also revealed reduction in CHIKV load. Fluorescence was barely detectable in the cells treated with 125 µM ZnAc compared to virus control (Fig. 5d). Similar effect of ZnAc treatment on CHIKV was also observed by flow cytometric analysis. Further, monitoring CHIKV E2 expression by flow cytometric analysis showed that >88% of the virus infected cells were positive for E2/FITC at 24 hpi, and after treatment with ZnAc only 0.59% cells showed E2 expression resulting in 99.3% viral reduction (Fig. 5f).

### *In silico* analysis

**Figure 6:**
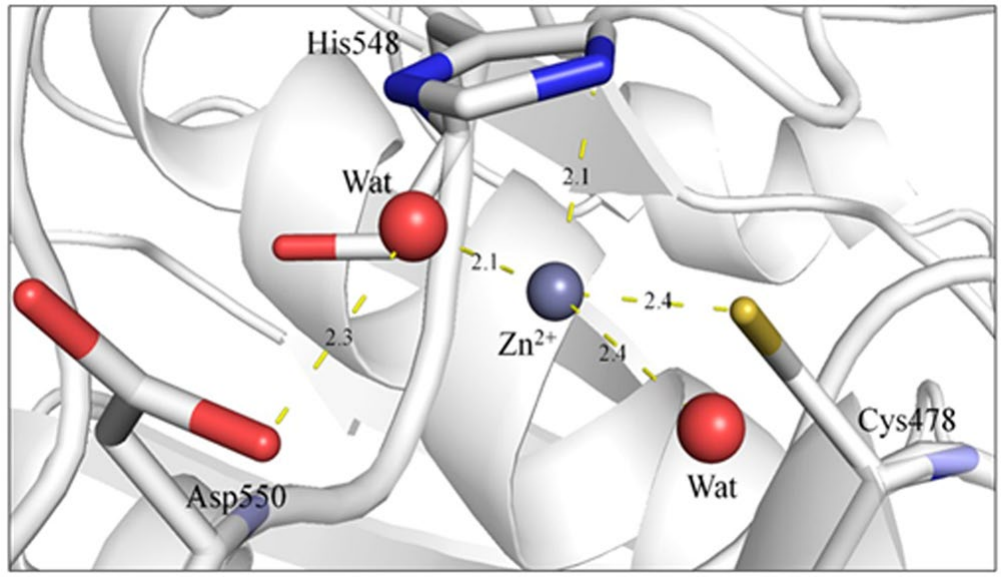
Zinc coordination in the active site of nsP2. The residues are shown in sticks and the coordinating water molecules are shown as red spheres. The coordinating distance in Å is shown with yellow sticks.

Next, the question was whether the zinc ion inhibition is specific to CHIKV nsP2pro. To justify the specificity of zinc ion against CHIKV nsP2pro, molecular docking was adopted. The MetSite server suggested that residues Cys478 and His548 of CHIKV nsP2 has the highest possibility of binding to zinc ion. The pocket near these residues was analysed for binding to zinc ion. Analysis of Zn binding showed that Zn coordinates with Cys478 and His 548 along with two water molecules. The water molecules coordinate with surrounding Asp550 (Fig. 6).

## Discussion

Effective countermeasures including development of promising therapeutics against CHIKV remain one of the important research challenges for the scientific community. Viral proteases are excellent target for development of antiviral therapeutics, due to their crucial role in viral polyprotein processing. Thus, as a pre-requisite for screening of small molecule inhibitors (SMIs) against CHIKV nsP2pro, we standardized large scale production of a soluble, catalytically active recombinant viral protease, with high yield and homogeneity in prokaryotic system. Further, we developed a reliable HTS assay with high sensitivity, selectivity and reproducibility to screen targeted compounds with anti-CHIKV nsP2 activity.

Among various expression systems, *E*. *coli* is one of the most widely used expression hosts, as it offers a simple, faster inexpensive way to obtain large quantities of recombinant proteins^25^. We used pET41a vector for cloning CHIKV nsP2pro, as it provides integrated advantage of high level expression, purification and detection using either GST or 6xHis tag. The GST moiety of this vector increases the stability and solubility of the protein as well as act as a chaperone to facilitate protein folding^26,27^. Typically, some nonbacterial proteins are produced in the form of insoluble inactive inclusion bodies, when overexpressed in *E*.*coli*. Therefore, we optimized the expression conditions through induction at lower temperature (18 °C) with lower concentration of inducer (0.5 mM IPTG) for overnight, which led to shifting of the expression to a soluble form, maintaining the biological activity of the protease.

Development of a HTS assay requires large quantity of active protease, which is difficult to achieve in shake flask due to inherent problem of batch to batch variation. No robust protocols for large scale production and purification of rCHIKV nsP2pro have been described so far. Therefore, HCDC batch fermentation was performed to produce large amount of biomass for purification of rCHIKV nsP2pro using 7 L volume bioreactor (with ~5 L working volume). The cultivation medium used for batch run was 2X LB with 1% glycerol. The components of the growth media are important for enhancement of product yield as well as reduction in by-product formations. Different cultivation medium like LB, TB and SB with some modifications were tried at small scale, results were satisfactory using simple 2XLB with 1% glycerol regarding overall yield of a bioactive enzyme. Glycerol reduces the acetate formation and also used as an antifoaming agent^28^. Final dry cell weight after the batch process was found to be increased more than 10 times when compared with that of the shake flask culture. This work yielded ~22 g/L of wet cell paste, which upon purification resulted in ~62 mg/L of GST-nsP2pro and 6 mg/L active untagged protease. The yield was superior to shake flasks and proved to be an efficient, easy to run, and inexpensive method for production of active protease.

The enzymatic activity of rCHIKV nsP2pro was further characterized by using fluorescence substrate. Fluorescence-based approaches have been widely used in assays for proteases and other drug targets mainly because of their high sensitivity. For development of HTS assay two different approaches have been used to measure protease activity i.e., FI and FRET based. The success of FI based assays remained ambiguous for alphaviruses^16^, though this was reported successful for flaviviruses^29,30^. In this study, Boc-AGG-MCA substrate was used in FI assay. Although, florescence unit of test reaction was found higher than the background signal but a proper enzyme curve with time was not obtained. Therefore, we tried similar type of substrates (Boc-AGG-AMC) with different fluorophore (AMC), but similar result was observed. Subsequently, FRET based substrate (DABCYL -RAGGYIFS-(E-EDANS)-NH_2_) was used with success. FRET based substrate are reported to be superior than FI based substrate for characterization of endopeptidases due to its low background signal^24^. The cleavage site preference of the CHIKV nsP2pro enzyme is nsP3-nsP4 > nsP1-nsP2 > nsP2-nsP3^9^. Since nsP3-nsP4 substrate is preferred for cleavage in *trans*^31^, we used this in our study. Further, mutated protease, C478A CHIKV nsP2pro used as a negative control was not able to cleave the substrate efficiently indicating that the C478 is crucial for nsP2pro activity.

Alphavirus nsP2 proteases are papain like cysteine protease having catalytic dyad consisting of conserved Cys and His^9,10^. The Cys residue acts as a nucleophile in the active site of the enzyme. Thus, the biochemical activity of the purified CHIKV nsP2pro was evaluated with generic irreversible (NEM) and reversible (MMTS) inhibitors, commonly used in biochemical studies involving thiol-dependent enzymes. As expected, the addition of sulfhydryl reagents such as MMTS and NEM strongly inhibited CHIKV nsP2 protease activity. Moreover, the addition of DTT or β-ME restored the enzyme activity inhibited by MMTS thereby confirming that Cys is required for the catalysis of polyprotein processing and that CHIKV nsP2pro is a cysteine protease^31^.

The optimal temperature for protease activity was found to be 22-28 °C which corresponds well to the appropriate environments for CHIKV growth in the vector. This corroborates with the fact that replication rate of CHIKV is higher in mosquito cells compared to mammalian cells^32,33^.

Further, the effect of different universal protease inhibitors was also tested. The enzyme was completely resistant to the inhibitors of serine proteases (PMSF, trypsin protease inhibitor I), aspartic proteases (pepstatin) and metalloproteases (EDTA). It was moderately inhibited by cysteine protease inhibitor (leupeptin) followed by chymostatin, which is reported to inhibit papain. Both untagged (nsP2pro) and tagged (GST-nsP2pro) protease were capable of hydrolysing the DABCYL -RAGGYIFS- (E-EDANS)-NH2 substrate. However, to avoid interference of fusion tag on protease activity, it is advantageous to remove fusion tag prior to use. Therefore, all the subsequent experiments were carried out using untagged nsP2pro. Kinetics of nsP2pro with and without His-tag has been recently studied with no interference on protease activity^31^.

Effect of different divalent cations like Ca^2+^, Cd^2+^, Cu^2+^, Hg^2+^, Mg^2+^, Ni^2+^ and Zn^2+^ were also studied on activity of nsP2pro. Out of these divalent cations Hg^2+^, Cu^2+^ and Zn^2+^ remarkably decreased protease activity. Zinc and its conjugates have been proven effective for inhibiting SARS-CoV replication *in vivo* as identified by inhibition of viral proteases in a biochemical assay^34^. Thus, we were interested in testing the influence of metal conjugated compound on CHIKV nsP2 protease activity. We first examined the ZnAc in our biochemical assay, which inhibited protease similarly like Zn^2+^. As ZnAc is FDA approved drug and is already used as therapeutics in humans^35^, therefore we further tested the efficacy of ZnAc to inhibit CHIKV on Vero cells. Interestingly, it was found to significantly inhibit CHIKV titer by more than 3 log. The mechanism of action of ZnAc has been successfully reported for Wilson’s disease, where it act on metallothionein, a family of cysteine-rich proteins^34^. Therefore, ZnAc might also inhibit the CHIKV cysteine protease in a similar manner. Further, docking studies showed that zinc has the highest possibility of binding to the catalytic dyad Cys and His at the active site. Similar zinc coordination sites are observed in the crystal structures of caspase-3 and mammalian NOS regulator dimethylarginine dimethylaminohydrolase. In caspase-3, the residues Cys163 and His121 along with two water molecules stabilize the Zn ion in the active site^36^. In the structure of the mammalian NOS regulator dimethylarginine dimethylaminohydrolase, the Cys273 and His172 along with two water molecules stabilize the Zn ion in the active site with Asp78 residue showing coordination with water molecules^37^. Therefore, this opens the scope for repurposing ZnAc and testing of other FDA approved metal conjugated drugs against CHIKV that can offer significant advantage with respect to fast track drug development.

The advantage of cell free biochemical assay include- no requirement of high containment facility (BSL-3/4), better accessibility of the compounds to the target, a well-defined mechanism of action, the possibility of developing inexpensive screens, the easy adaptability to newer technologies, amenability to miniaturization, and more ready automation. Thus, a robust and sensitive method for identification of nsP2protease inhibitors in a HTS format was developed with the goal of identifying compounds with anti-CHIKV drug activity. We have optimized the signal window (S/B > 5) of the assay by determining the most favourable assay parameters such as pH, temperature, salt tolerance, assay buffer, assay stability and volume. Finally, the optimized assay was statistically validated for use in a FRET-based HTS biochemical assay for screening SMIs with low variability. Thus, a reliable and reproducible assay have been developed with Z’-factor 0.67 ± 0.054 and intra-plate, inter-plate, day to day variability of CV < 10%. This platform provides an opportunity for identification of potent nsP2pro inhibitors for subsequent use as lead molecule, which would potentially provide drugs capable of treating CHIKV infection in the near future. Further, identification of zinc and its conjugate (ZnAc) as an inhibitor of CHIKV nsP2pro in this study provides an opportunity for repurposing of these kind of approved drugs and mineral supplements for antiviral therapy against CHIKV infection.

## Methods

### Virus and cell line

An Indian isolate of Chikungunya virus, DRDE-07 (GenBank Accession number: EU372006) was propagated in C6/36 cells (*Aedes albopictus* larval cells) using standard virus adsorption techniques. The Chikungunya virus was handled in a biosafety level (BSL) 3 laboratory. Vero cells (African green monkey kidney cells) were used for viral quantification and antiviral assay^38^.

### Cloning and expression of nsP2pro

Viral RNA was extracted from CHIKV infected C6/36 cells using QIAamp viral RNA mini kit (Qiagen, Germany) according to manufacturer’s instructions. The genomic region coding for nsp2pro was amplified employing the designed primers (FP: 5′CCCATGGAGGATACATTCCAAAATAAAGCC3′ and RP: 5′TGCTCGAGACATCCTGCTCGGGTGAC3′) by two-step RT-PCR using M-MLV RT and Pfu DNA polymerase (Promega, USA). It was cloned into *NcoI* and *XhoI* sites of the pET41a expression vector. The plasmid was transformed into *E*.*coli* BL21 (DE3) and transformed cells were cultured at 37 °C in Luria Bertani (LB) medium containing 50 µg/ml kanamycin. Expression was induced at OD_600_ = 0.6 with 1 mM isopropyl-β-D-thiogalactopyranoside (IPTG), culture was further incubated for 4 h at 37 °C and expression profile was studied by SDS-PAGE.

### Shake flask cultivation and purification

Prior to large-scale cell growth and purification, rCHIKV nsP2pro fusion protein was produced on a small scale to assess its solubility. The amount of fusion protein in the soluble fraction of the crude cell lysate was compared with the total amount of fusion protein in the cells by SDS-PAGE. In order to shift expression to a soluble form two simple strategies were adopted, viz., lowering the concentration of inducer (0.5 mM, and 0.1 mM IPTG) and induction at lower temperature (30 °C, 27 °C and 18 °C) for overnight. After optimization, shake flask cultivation was carried out in 1 L LB medium in the presence of 50 µg/ml kanamycin. At mid-log phase, the culture was induced with 0.5 mM IPTG and grown for overnight at 18 °C before harvesting and purified using Ni^2+^-NTA affinity chromatography. Following purification, the peak fractions were pooled, desalted and enterokinase digestion was carried out. The concentration of the purified protein was estimated by bicinchoninic acid (BCA; Sigma, USA). Western blot analysis using rabbit 1:3000 anti-nsP2 polyclonal (a kind gift from Dr. Soma Chattopadhyay, Institute of Life Sciences, Bhubaneswar), 1:1000 anti-His monoclonal (Qiagen, Germany) or 1:1000 anti-GST monoclonal (Qiagen, Germany) antibody was used to confirm the expression of the rCHIKV nsP2pro. GST protein was also purified for control reaction using protocol as described previously^39^. Further, mutation of the conserved catalytic residue Cys478 to Ala was done for using nsP2pro Cys478Ala mutant as a negative control in FRET proteolytic assay. The site-directed mutagenesis experiment was carried out using mutagenic primer for Cys to Ala mutation (5′TAAAGCCAACGTT**GCT**TGGGCTAAGAGCTTGG-3′ and 5′CCAAGCTCTTAGCCCA**AGC**AACGTTGGCTTTA-3′). Cloning was carried out in pET28c vector and mutation of Cys478Ala in the isolated plasmid was confirmed by DNA sequencing. Mutated protease was expressed, purified and detagged using procedure similar to that of wild type CHIKV nsP2pro.

### Cultivation in Bioreactor

Prior to large-scale cultivation, different media like LB, TB and SB with some modifications were tried at small scale for enhancing protein production. After optimization, in bioreactor (BioFlo 3000 bioreactor, New Brunswick Scientific, USA), cultivation was carried out in batch mode using Biocommand Bioprocessing software (New Brunswick Scientific, USA) to regulate and record all batch run using 2XLB with 1% glycerol. To inoculate each batch run, 1 ml of stock culture of rCHIKV nsP2pro *E*. *coli* cells was inoculated into 1 L flask containing 100 ml LB and grown at 37 °C for 8 h at 200 rpm. Further about 4.5 ml of this grown culture was aseptically inoculated into 5 L flask containing 450 ml sterile modified LB medium (2XLB with 1% glycerol) with kanamycin and grown overnight at 37 °C with continuous shaking at 200 rpm. Thereafter, 450 ml of overnight grown culture was aseptically inoculated into a 7 L capacity bioreactor, containing 4.5 L sterile medium (2XLB with 1% glycerol) with kanamycin. The agitation, pH, temperature and air flow rate were initially set to 200 rpm, 7.0, 37 °C and 5 Lmin^−1^ respectively. The pH was controlled at 6.8–7.2 using 25% ammonium hydroxide, DO was maintained at 30% of saturation and was controlled by a dissolved oxygen cascade of agitation up to 600 rpm and pure oxygen supply as required. Foaming was prevented by use of antifoam agent during the run. Culture was grown till the mid log phase for five hours post inoculation and at an OD_600_ of 20 the culture was induced with 0.5 mM IPTG. During induction process all set parameters were kept identical except the temperature, which ‘was then decreased and maintained at 18 °C. Samples were collected during the course of run for the measurement of optical density (OD_600_), dry-cell weight (DCW) and wet-cell weight (WCW) analysis. The fermentation and downstream processing were carried out in a current Good Manufacturing Practices (cGMP) compliant facility with class 10000 and 1000 respectively.

### Purification of CHIKV nsP2pro

The bacterial pellet was resuspended in lysis buffer (50 mM NaH_2_PO_4_, 300 mM NaCl, 10 mM imidazole, pH 8.0) in 1:20, w/v and incubated on ice for 30 min. Cells were disrupted using dyno mill (Willy A. Bachofen AG Maschinenfabrik, Switzerland) and the clarified soluble fraction was collected by centrifugation at 10,000 × g at 4 °C for 30 min. This was further filtered by 0.45-μm membrane to remove any particulates and applied on the column containing pre-equilibrated Ni^2+^-NTA superflow resin (Qiagen, USA) connected with an AKTA Explorer (GE Healthcare Life Sciences, Sweden). The column was first washed with wash buffer 1 (50 mM NaH_2_PO_4_, 300 mM NaCl, 20 mM imidazole, pH 8.0) followed by wash buffer 2 (50 mM NaH_2_PO_4_, 300 mM NaCl, 50 mM imidazole, pH 8.0). The protein fraction was eluted in elution buffer (50 mM NaH_2_PO_4_, 300 mM NaCl, 250 mM imidazole, pH 8.0) and analyzed by 10% SDS-PAGE. Following purification, the peak fractions were pooled, desalted using PBS as exchange buffer, untagged, concentration of the purified protein was estimated and stored at 4 °C.

### MALDI-TOF/MS analysis

For mass spectrometry analysis of rCHIKV nsP2pro fusion protein, samples were run on an SDS-PAGE gel, and the corresponding band of protein was excised and processed using a protocol as described earlier^39^. The spectra were evaluated using the Flex Analysis Software (Bruker Daltonics, USA). The MS spectra obtained was submitted to MASCOT search engine via Bio tools versions 3.1. The National Center for Biotechnology Information (NCBI) protein database was used for search.

### Protease assay

Initially, FI based substrates (Boc-AGG-MCA, Boc-AGC-AMC) were used in the protease assay, but protease activity could not be detected with these substrates and thus, FRET-based substrate was employed (DABCYL -RAGGYIFS- (E-EDANS)-NH_2_ (Biolink, India). This substrate at different concentration ranging from 0.5–50 µM in assay buffer (20 mM Bis-Tris-Propane, pH 8.0) was added to Nunc 96 Well black plates with 1 µM protease diluted in the same buffer in triplicates. For kinetics measurements, fluorescence readings were measured at 5 min interval for 15 h using Synergy H1 Multimode plate reader with Gen5 software (BioTek, USA) at an excitation wavelength (λex) of 340 nm and an emission wavelength (λem) of 490 nm. The cleavage reactions were initially performed at the different time points from 0 to 15 h, and relative fluorescence unit (RFU) was measured by subtracting background fluorescence (NC). The time point of 6 h was later selected, which is in the linear phase of the cleavage activity, for subsequent experiments. To determine the amount of fluorophore released, a (E-EDANS)-NH_2_ standard curve was plotted.

### Optimal reaction conditions and the effects of protease inhibitors

To further characterize the biochemical properties of nsP2pro, the protease activity was measured under varying reaction conditions, such as pH (5.5–11), temperature (4°–48 °C), salt content (50 mM–300 mM NaCl), or following treatment with various cysteine, serine, aspartyl or metalloprotease inhibitors (leupeptin, trypsin protease inhibitor I, chymostatin, pepstatin, PMSF, EDTA; Sigma, USA). Further, we examined the effects of various divalent cations on the protease activity by supplementing the reaction with 2 mM of HgCl_2_, CuCl_2_, CdCl_2,_ NiCl_2,_ MgCl_2_, CaCl_2_, or ZnCl_2_. Finally, known inhibitors of cysteine protease, NEM and MMTS were examined to determine their effects on the activity of the nsP2pro.

### High-throughput screening protease assay

The protease activity was measured in black 96-well plate (100 µl) in 48 replicates of each positive and negative control. Finally, to conduct a rigorous evaluation of the assay variability and plate uniformity, calculation of the Z’ factor, coefficient of variation, signal-to-background ratio (S/B) and signal-to-noise ratio (S/N) was carried out^24^.

### Inhibition assay (metal-conjugated compound)

The anti-CHIKV nsP2pro activity of ZnAc (2 mM, Sigma, USA) was evaluated by biochemical assay in a 100 µl reaction volume. Fluorescence was measured by monitoring the release of EDANS every 5 min up to 6 h. The percent inhibition value was calculated from the RFUs obtained in the presence and absence of ZnAc.

### Antiviral assay

To evaluate the ability of ZnAc to block CHIKV replication, the cytotoxic effect of the ZnAc was determined by using MTT (3-(4,5-dimethylthiazol 2-yl)-2,5-diphenyltetrazolium bromide) assay in Vero cells. Vero cells were seeded into 96-well culture plates at cell density of 1 × 10^4^ cells per well and allowed for overnight incubation for cell attachment. Serial two-fold dilution of compound was prepared in MEM from 8 mM to 3.9 µM. The plates were incubated in 37 °C, 5% CO_2_ for 48 hours. After 48 hours incubation, MTT solution was added into each well and incubated for 3 hour at 37 °C. The absorbance was measured at 570 nm and MNTD was determined. Further, for inhibition assay, Vero cells were infected with CHIKV at multiplicity of infection (MOI) of 1 except for the cell control. ZnAc was added 2 hours following viral adsorption in concentration ranging from 125 µM to 3.9 µM. The plates were incubated in 37 °C, 5% CO_2_ for 48 h. The infected supernatant was collected at 48 h post infection (hpi) for quantitation of infectious virus by plaque assay and cell viability was examined in the remaining cells through the MTT test. To test the reduction of viral load after ZnAc treatment; plaque assay, immunofluorescence assay and reduction of CPE were carried out as described earlier^38^. For flow cytometric analysis, cells were infected with CHIKV at MOI of 0.1 in a 75 cm2 cell culture flask followed by treatment with 100 µM ZnAc in a post treatment mode. Infection was allowed to proceed for 24 hpi. The mock Vero cells and CHIKV infected cells with or without ZnAc were detached by trypsinization. Cells were harvested, fixed (chilled methanol for 15 min on ice), permeabilized (0.1% Triton-X 100/0.5% BSA/PBS for 10 min only) and blocked (3% BSA/PBS for 30 min at RT). Cells were then probed with primary antibody CHIKV E2 mAb^40^ in 3% BSA/PBS and incubated for 30 min at RT followed by washing the cells thrice by centrifugation at 500 g for 5 min with ice cold PBS. Further secondary antibody anti-mouse FITC in 3% BSA/PBS was added and incubated for 30 min at RT in the dark. Washing was repeated as above and cells were resuspended in ice cold 3% BSA/PBS and acquired by FACSCaliburTM flow cytometer and analyzed by CellQuest Pro software (BD Biosciences, USA).

### *In silico* studies

The MetSite server (http://bioinf.cs.ucl.ac.uk/structure/) was used for the prediction of zinc binding sites in CHIKV nsP2. The sequence of the protein was given as input to the server. The crystal structure of CHIKV nsP2pro (PDB ID 4ZTB) was used for analysis to predicted zinc bind site. The zinc ion and water molecules were placed in the structure using coot.

### Statistical analyses

For enzyme kinetics, initial velocities were calculated using the linear regression function in the Microsoft Office excel software. Data were analyzed and plotted using Michaelis-Menten equation with GraphPad Prism to obtain kinetic parameters. To evaluate the robustness of HTS protease assay, Z’ factor, CV, S/B and S/N were calculated. All the assays were performed in triplicate and results were graphed, with error bars indicating the SD. Inhibition assay and antiviral data were analyzed using student’s t-test and p value < 0.05 was considered statistically significant.

### Data availability statement

The data generated or analysed during the study are included in this article and its supplementary information file.

## Electronic supplementary material

Supplementary Information
