# Supplementary material for: Development of nsP2 protease based cell free high throughput screening assay for evaluation of inhibitors against emerging Chikungunya virus
Source: Sci Rep. 2018 Jul 17;8:10831. doi: 10.1038/s41598-018-29024-2 (PMC6050329; doi:10.1038/s41598-018-29024-2)
Supplement: Supplementary file 1 — Supplementary Information [file 41598_2018_29024_MOESM1_ESM.docx]

**Development of nsP2 protease based cell free high throughput screening assay for evaluation of inhibitors against emerging Chikungunya virus**

**Amrita Saha^1^, Badri Narayan Acharya^2^, Raj Priya^1^, Nagesh K. Tripathi^3^, Ambuj Shrivastava^1^, M. Kameswara Rao^4^, Pooja Kesari^5^, Manju Narwal^5^, Shailly Tomar^5^,** **Sameer S. Bhagyawant^6^, Manmohan Parida^1^, Paban Kumar Dash^1*^**

*^1^Virology Division; ^2^Synthetic Chemistry Division; ^3^Bioprocess Technology Division; ^4^Pharmacology & Toxicology Division, Defence Research & Development Establishment, Gwalior 474002, India*

*^5^Department of Biotechnology, Indian Institute of Technology Roorkee, Roorkee 247667, India*

*^6^School of Studies in Biotechnology, Jiwaji University, Gwalior, India*

*Corresponding author at:

Dr. Paban Kumar Dash

Virology Division, Defence Research & Development Establishment (DRDE), Jhansi Road, Gwalior 474002, M.P, India

E-mail address: [pabandash@drde.drdo.in](mailto:pabandash@drde.drdo.in), [pabandash@rediffmail.com](mailto:pabandash@rediffmail.com)

**Figure S1:** Purification and characterization of rCHIKV nsP2pro. (**a**) Shake flask culture was purified under native condition using IMAC (UN: uninduced; FT: flow through; CL: cleared lysate; W1-W2: wash fractions; E1–E9: eluate fractions; M_1_: marker protein (#SM0661 Fermentas, USA). Coomassie-stained 12.5 % SDS-PAGE gel showing 29 kDa GST protein which was purified under native condition to be used as a control to confirm digested GST moiety size and as a negative control for protease assay. CHIKV nsP2pro after cleavage reaction (shake flask culture). (**b**) Media optimization in shake flask culture using LB broth, LB+glycerol, 2XLB, 2XLB+glycerol, TB and SB. Batch culture was carried out using 2XLB+1%glycerol and rCHIKV nsP2pro was purified under native condition on a Ni^2+^-NTA Superflow column connected to an Akta purification system (E0–E7: eluate fractions). SDS-PAGE gel showing: (1) eluted fractions pooled and passed from 50 kDa cutoff membrane; (2) untagged nsP2pro (39 kDa); (3) nsP2pro fusion protein (FP) and (4) untagged nsP2pro (50 µg each). (**c**) The western blot analysis of purified CHIKV nsP2pro fusion protein using anti-His (1) or anti-GST monoclonal antibody (2). Analysis of endoproteolytic cleavage of the GST moiety from the CHIKV nsP2pro fusion protein using enterokinase enzyme (3). Specificity of enterokinase digested CHIKV nsP2pro was confirmed by immunodetection, probed with polyclonal nsP2 rabbit sera: digestion reaction (4) and (5) purified untagged CHIKV nsP2pro (M_2_:marker protein, #C1992 Sigma, USA). Stability studies: (**d**) Stability study of CHIKV nsP2pro fusion protein stored at –80 °C, over a 12 month period. Protein was purified, dialysed, detagged and concentration was determined by the BCA method using a BSA standard. (**e**) Relative activity of CHIKV nsP2pro was also determined by taking activity at 0 month as 100%. Values are the average of two experiments and the error bars represent the standard deviation.


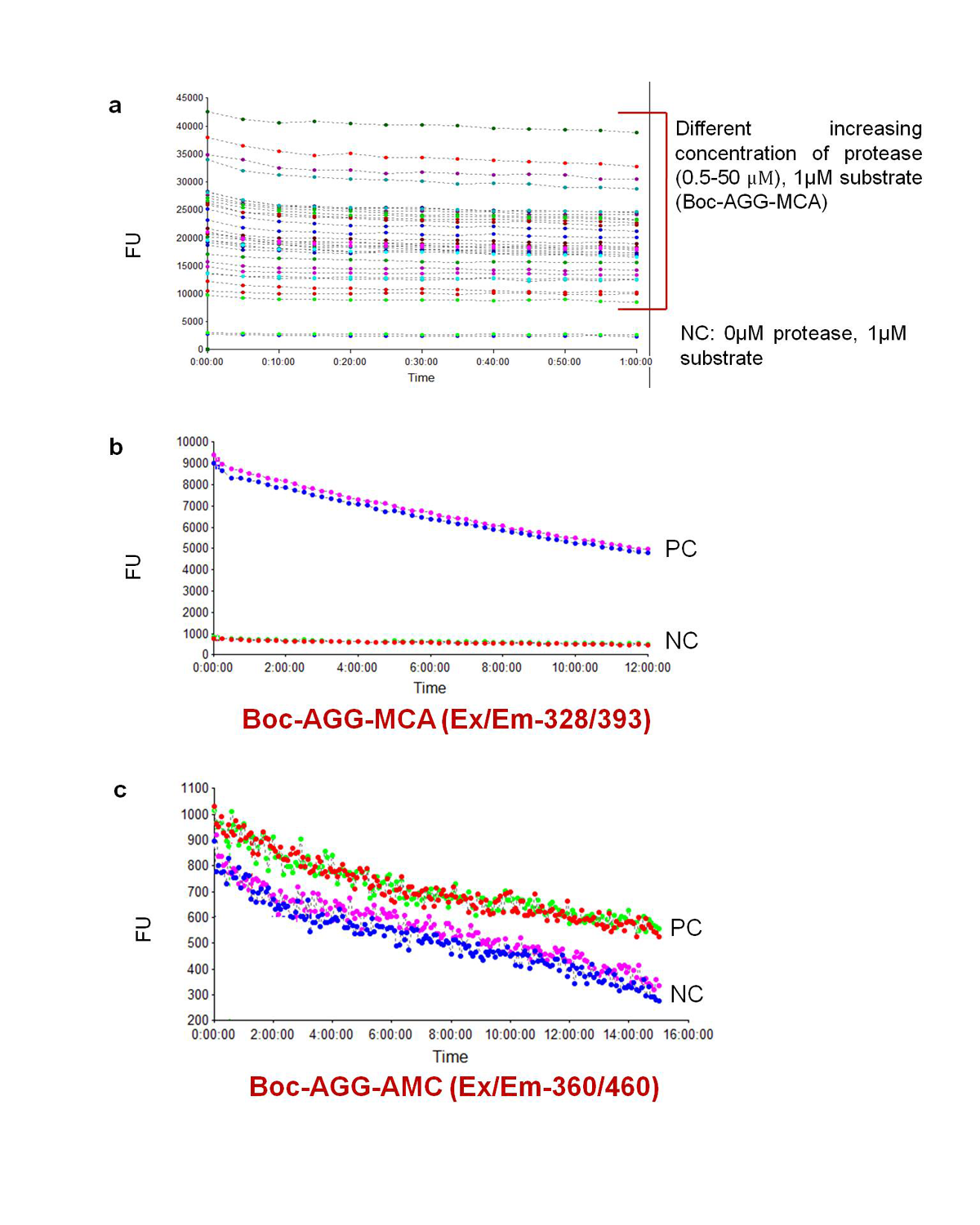


**Figure S2:** In fluorescence intensity (FI) based assays, the fluorescent group (MCA or AMC) is covalently linked to peptide substrate (AGG) making it non-fluorescent. Following reaction, protease cleaves the substrate, freeing the fluorescent group, and the reaction is monitored as an increase in fluorescence signal. Real-time profile of FI based protease assay: (**a** and **b**) using Boc-AGG-MCA and (**c**) Boc-AGG-AMC substrate. Although, fluorescence unit (FU) of test reaction (PC) was found higher than the background signal (NC) but increase in fluorescence signal with time i.e., a proper enzyme saturation curve was not obtained (**b** and **c**: 100 µl reaction was carried out using 20 mM BisTrisPropane pH 8.0 as assay buffer at 25 °C with 1 µM respective substrate for 12-15 h).

**Figure S3:** Purification and characterization of C478A CHIKV nsP2pro. (**a**) Shake flask culture was purified under native condition using IMAC (CL: cleared lysate; FT: flow through; W1-W2: wash fractions; E1–E11: eluate fractions; M: marker protein (#SM0661 Fermentas, USA); 1: dialysed and detagged C478 CHIKV nsP2pro (39 kDa). (**b**) FRET protease cleavage reaction was performed in the presence of C478A CHIKV nsP2pro, which did not showed change in the fluorescence intensity (1 µM C478A CHIKV nsP2pro or wild type CHIKV nsP2pro, 3 µM substrate, 20 mM BisTrisPropane pH 8.0 as assay buffer in 100 µl reaction volume at 25°C for ~15 h). Real time profile monitored by fluorescence plate reader shows very low level processing, which was almost negligible compared to wild type protease.
